# Implant decontamination with phosphoric acid during surgical peri-implantitis treatment: a RCT

**DOI:** 10.1186/s40729-017-0091-5

**Published:** 2017-07-17

**Authors:** Diederik F. M. Hentenaar, Yvonne C. M. De Waal, Hans Strooker, Henny J. A. Meijer, Arie-Jan Van Winkelhoff, Gerry M. Raghoebar

**Affiliations:** 1 Department of Oral and Maxillofacial Surgery, University of Groningen, University Medical Center Groningen, PO Box 30.001, 9700 RB Groningen, The Netherlands; 2 Center for Dentistry and Oral Hygiene, University of Groningen, University Medical Center Groningen, Groningen, The Netherlands; 3 Department of Medical Microbiology, University of Groningen, University Medical Center Groningen, Groningen, The Netherlands

**Keywords:** Peri-implantitis, Decontamination, Dental implants, Surgery, Microbiology

## Abstract

**Background:**

Peri-implantitis is known as an infectious disease that affects the peri-implant soft and hard tissue. Today, scientific literature provides very little evidence for an effective intervention protocol for treatment of peri-implantitis. The aim of the present randomized controlled trial is to evaluate the microbiological and clinical effectiveness of phosphoric acid as a decontaminating agent of the implant surface during surgical peri-implantitis treatment.

**Methods:**

Peri-implantitis lesions were treated with resective surgical treatment aimed at peri-implant granulation tissue removal, bone recontouring, and pocket elimination. Fifty-three implant surfaces in 28 patients were mechanically cleaned and treated with either 35% phosphoric etching gel (test group) or sterile saline (control group). Microbiological samples were obtained during surgery; clinical parameters were recorded at baseline and at 3 months after treatment. Data were analyzed using multi-variable linear regression analysis and multilevel statistics.

**Results:**

Significant immediate reductions in total anaerobic bacterial counts on the implant surface were found in both groups. Immediate reduction was greater when phosphoric acid was used. The difference in log-transformed mean anaerobic counts between both procedures was not statistical significant (*p* = 0.108), but there were significantly less culture-positive implants after the decontamination procedure in the phosphoric acid group (*p* = 0.042). At 3 months post-surgery, 75% of the implants in the control group and 63.3% of the implants in the test group showed disease resolution. However, no significant differences in clinical and microbiological outcomes between both groups were found.

**Conclusions:**

The application of 35% phosphoric acid after mechanical debridement is superior to mechanical debridement combined with sterile saline rinsing for decontamination of the implant surface during surgical peri-implantitis treatment. However, phosphoric acid as implant surface decontaminant does not seem to enhance clinical outcomes on a 3-month follow-up.

**Trial registration:**

Netherlands National Trial Register, NTR5185 (www.trialregister.nl)

## Background

Triggered host defense responses initiate inflammation of the peri-implant soft tissue (peri-implant mucositis), which can lead to loss of peri-implant supporting bone (peri-implantitis), and eventually, result in implant failure [1]. An increasing prevalence of peri-implantitis has been described in recent literature [2], with current incidence ranging from 1 to 47%. A non-linear, accelerating pattern of progress is suggested for the majority of cases, with an occurring onset within 3 years of function [3]. As for periodontal disease, the presence of micro-organisms is an important factor for the development of an inflammatory response in peri-implant tissue [4]. In order to effectively treat the peri-implant inflammation, disruption of microbial adhesion and reduction of biofilm accumulation on the implant surface is probably of eminent importance.

A number of mechanical interventions (e.g., abrasive air powder, teflon curettes, ultrasonic devices) and chemical agents (e.g., chlorhexidine, hydrogen peroxide) solely or in combination have been described as methods for implant surface decontamination in both in vivo and in vitro studies, in both a surgical and non-surgical setting ([5–12]). According to different reviews on in vivo and in vitro mechanical debridement [13–17], a gold standard mechanical debridement regimen still does not exists. Possibly, the implant surface roughness and screw-shaped design of dental implants may compromise an effective mechanical intervention. Therefore, the additional use of chemical agents for implant decontamination may be advocated.

Antimicrobial solutions have been studied in different clinical studies [9, 10, 18, 19]. No superior clinical effectiveness has been shown in a single study for a specific chemical decontamination protocol (for reviews see [17, 20, 21]). However, studies using acids at low pH (<2) have shown potentially beneficial antiseptic effects [22–29]. Especially, results on decontamination with phosphoric acid might be promising. Wiltfang et al. [27] showed that surface decontamination with phosphoric acid (pH 1) in a surgical treatment protocol resulted in complete elimination of the bacterial microflora. Also, results of a short-term clinical trial by Strooker et al. [26] showed an instant greater reduction of colony-forming units on the implant surface when using phosphoric etching gel (pH 1). In addition, animal studies [30, 31] showed re-osseointegration and direct bone-to-implant contact when acids were used. Therefore, phosphoric acids might be considered a potentially feasible decontaminating agent.

Thus far, the use of phosphoric acid etching gel as decontaminating agent has not been evaluated in a randomized controlled trial. The aim of the present randomized controlled trial is to evaluate the short-term microbiological and clinical effectiveness of 35% phosphoric etching gel as a decontaminating agent of the implant surface during resective surgical treatment of peri-implantitis.

## Methods

### Trial design

The present study is a double-blind randomized controlled trial evaluating the effect of 35% phosphoric etching gel (test group) compared to the effect of saline (control group) for implant surface decontamination combined with mechanical debridement during surgical peri-implantitis treatment. Patients were randomly assigned to the test or control group using a one-to-one allocation ratio. The study has been conducted in full accordance with the World Medical Association Declaration of Helsinki (version 2008) and was approved by the Institutional Review Board of the University Medical Center Groningen, the Netherlands (METc2013.005). Written informed consent was obtained from all participants before entering the trial. Clinical trial registration was done at the Netherlands National Trial Register (http://www.trialregister.nl, trial number NTR5185). The CONSORT guidelines for reporting a clinical trial were followed.

### Participants

Patients participating in this study were consecutively selected from the patient populations of the Center of Dentistry and Oral Hygiene and the Department of Oral and Maxillofacial Surgery of the University Medical Center Groningen, Groningen, The Netherlands, from October 2012 to April 2014.

Adult patients with at least one endosseous implant with clinical and radiographical signs of peri-implantitis were included. Peri-implantitis was defined as a loss of marginal bone ≥2 mm in combination with bleeding and/or suppuration on probing and a peri-implant probing depth ≥5 mm [32]. Implants had to be in function for at least 2 years.

Exclusion criteria were:

- Contraindications for the surgical procedures;
- A history of local radiotherapy to the head and neck region;
- Pregnancy and lactation;
- Uncontrolled diabetes;
- Systemic use of antibiotics within 3 months before inclusion;
- Long-term use of anti-inflammatory drugs;
- Incapability of performing basal oral hygiene measures as a result of physical or mental disorders;
- Uncontrolled periodontitis (PPD >5 mm);
- Implants with bone loss exceeding two thirds of the length of the implant or implants with bone loss beyond the transverse openings in hollow implants;
- Implant mobility;
- Implants at which no position could be identified where proper probing measurements could be performed;
- Previous surgical treatment of the peri-implantitis lesions.

### Interventions

The study protocol was based on the study protocols of two previous studies evaluating the decontaminating effect of chlorhexidine during surgical peri-implantitis treatment [10, 32] and is briefly described below.

Within 1 month before surgical treatment, all patients received extensive oral hygiene instructions and mechanical non-surgical debridement of implants and remaining dentition using hand instrumentation and/or an ultrasonic device. Immediately before surgical treatment screw-retained suprastructures were removed. In order to obtain an optimal overview of the peri-implant area during surgery, prior to the procedure, only screw-retained suprastructures were removed. Cemented single crowns or bridges on mesostructures were left in place to prevent any damage to these structures. Directly after surgery, the screw-retained suprastructures were placed back. Cemented single crowns or bridges on mesostructures were left in place to prevent any damage to these structures. Vertical releasing incisions extending into the alveolar mucosa were placed using a surgical blade (no. 15), and full thickness mucoperiosteal flaps were raised buccally and lingually. Flaps were designed to allow optimal access to the peri-implant bone defect. Granulation tissue was removed using titanium curettes (Gracey; Hu-Friedy®, Chicago, IL, USA). The implant surfaces were mechanically cleaned using titanium curettes and gauzes and cotton pellets soaked in saline. Next, the patients were randomly allocated to either the test or control group. Subsequently, implants were cleaned with either local application of 35% phosphoric acid gel (pH 1) for 1 min (Temrex gel, Temrex, Freeport, NY, USA) (test group) or by rinsing with an abundant amount of sterile saline for 1 min (control group). Care was taken to apply the phosphoric etching gel precisely on the implant surface using a syringe with a small tip. During 1 min, the etching gel was continuously rubbed on to the implant surface with a small brush. In both groups, the intervention continued with rinsing of the implant surface with an abundant amount of sterile saline for 1 min.

Angular bony defects were eliminated, and bone was recontoured using a rotating round bur under saline irrigation. Mucosal flaps were apically positioned and firmly sutured (Vicryl Plus® 3-0; Ethicon Inc., Somerville, NJ, USA), and suprastructures were re-positioned. For both control and test group, surgery was followed by 2 weeks of mouth rinsing with 0.12% CHX + 0.05% CPC without alcohol two times daily for 30 s. Sutures were removed after 2 weeks. Follow-up visits were scheduled after 3 (T_3_) months. Patients were all surgically treated by one experienced oral and maxillofacial surgeon (GR).

### Outcomes

#### Primary outcome variable

The primary outcome variable was the difference in anaerobic bacterial load of the implant surface before and after mechanical and chemical debridement and decontamination. After flap deflection and granulation tissue removal, a sample was obtained from the implant surface by rubbing a sterilized brush (Microbrush® International, Grafton, WI, USA) across the implant surface (T_pre_). A second sample was obtained after mechanical debridement, decontamination of the implant surface with the test or control substance, and subsequent rinsing with sterile saline (Tpost). After sampling, the top part of the brush was cut off and collected in a vial containing reduced transport fluid [33]. From every implant presenting peri-implantitis, separate samples were obtained. All microbiological samples were processed within 24 h [34]. The total anaerobic bacterial load and the presence and numbers of the periodontal pathogens [35] *Aggregatibacter actinomycetemcomitans*, *Porphyromonas gingivalis*, *Prevotella intermedia, Tannerella forsythia*, *Fusobacterium nucleatum*, *Parvimonas micra*, and *Campylobacter rectus* were determined by laboratory technicians who were blind to treatment allocation.

#### Secondary outcome variables

Secondary outcome variables were percentage of sites with bleeding on probing (% sites BoP), percentage of sites with suppuration on probing (% sites SoP), mean probing pocket depth (mean PPD), and microbial composition of the peri-implant sulcus. Measurements were performed before (pre) treatment (baseline, T_0_) and at 3 months (T_3_) after surgery by one and the same examiner (DH) who was blind to treatment allocation. Peri-implant pocket depth was measured at four sites per implant (mesial, buccal, distal, and lingual) using a pressure sensitive probe (KerrHawe Click Probe®, Bioggo, Switzerland) (probe force of 0.25 N). Bleeding and suppuration were scored up to 30s after pocket probing. Microbiological peri-implant sulcus samples were collected from each implant with peri-implantitis using four sterile paperpoints per implant. Paperpoints were collected in a vial containing RTF and were analyzed in the same manner as the intra-operative samples. Outcome variables were total anaerobic bacterial load and the presence and numbers of the periodontal pathogens *A. actinomycetemcomitans*, *P. gingivalis*, *P. intermedia*, *T. forsythia*, *F. nucleatum*, *P. micra*, and *C. rectus*.

### Randomization

Fourteen notes with the word “phosphoric acid” and 14 notes with the word “saline” were put into 28 identical, sequentially numbered, non-transparent envelopes according to a randomization list generated by a computer program. The envelopes were irreversibly sealed. During the surgical procedure, after flap deflection and mechanical cleansing, the surgeon temporarily left the operating room. The surgical assistant opened an envelope and prepared the materials as needed according to the information on the note. A third person (YDW) performed the decontamination procedure according to group allocation. The materials were removed, and the surgeon continued the surgical procedure. The researcher (performing the clinical measurements, DH) was blind to treatment allocation and did not have access to the randomization code until the end of the research period.

### Statistical methods

#### Sample size

Sample size was based on the microbiological data from a previous study evaluating the effect of implant surface decontamination with a chlorhexidine solution versus a placebo solution [10]. The decontaminating effect of phosphoric acid was expected to be similar to the decontaminating effect of chlorhexidine (reduction in log-transformed mean anaerobic bacterial load = 4.21 (chlorhexidine group) versus 2.77 (placebo group), SD = 2.12). Assuming a two-sided two sample *t* test with a significance level (α) of 0.05 and a power (β) of 80% required a sample size of 34 implants. A 20% compensation for dropouts was taken into account (34/0.8 = 42.5 implants). Based on a previous study [10], it was expected that not all baseline microbiological samples would yield a detectable number of cultivable bacteria ([10], 19 out of 79 = 24% of samples showed no bacterial growth). Because “negative” samples cannot be used to determine a decontaminating effect, the sample size was compensated for these potential unusable samples (24%), yielding a sample size of 56 implants (42.5/0.76). According to the assumption that each patient has on average more than two implants with peri-implantitis [10], a sample size of 28 patients was chosen (56/2, 14 patients per group).

#### Statistical analysis

For the analysis of the primary outcome variable and the secondary *microbiological* outcome variable linear regression analysis was performed. The implant was taken as the statistical unit. Total anaerobic bacterial loads at baseline (T_pre_ and T_0_) were distributed normally after logarithmic transformation. Baseline values were included in the regression model. For the comparison of the number of culture-positive implants after the decontamination period, the chi-square test was used. The secondary *clinical* outcome variables were analyzed using a two-level hierarchical random intercepts model. The two levels of analysis were implant level and patient level. With the crude analysis, the effect of the intervention was determined, while controlling for baseline value. Because a previous study [9] has shown that mean bone loss at baseline and smoking are prognostic indicators for the outcome of resective peri-implantitis treatment, these factors were additionally included in the model (adjusted analysis).

Descriptive data and data regarding the microbiological outcome variables were analyzed using IBM SPSS Statistics 22 Version 22.0 (IBM Corp. Armonk, NY: IBM Corp.). Multilevel models were analyzed using MLwiN version 2.12 (Centre for Multilevel Modeling, University of Bristol, Bristol, UK).

## Results

**Fig. 1:**
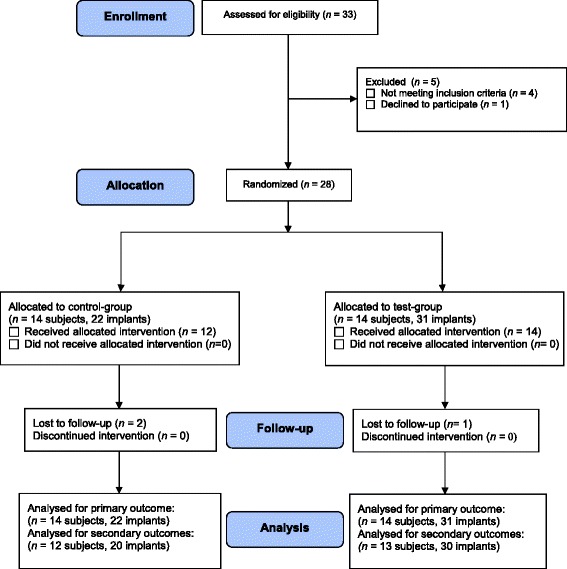
Flow diagram

The progress of patients throughout the different phases of the study is illustrated in Fig. 1. Table 1 depicts the baseline demographic patient and implant characteristics. The included patients had a total of 128 implants of which 53 implants showed signs of peri-implantitis. Different implant brands and types with different implant surfaces were present, including Straumann (Straumann AG, Basel, Switserland; SLA® and SLActive® surface), Nobel Biocare (Nobel Biocare AB, Göteborg, Sweden; TiUnite® surface), Biomet 3i (Biomet Inc., Warsaw, Indiana, USA; OSSEOTITE® surface), Frialit-2, (Dentsply Friadent, Mannheim, Germany; FRIADENT® plus surface), and Pitt-Easy (Sybron Implant Solutions GmbH, Bremen, Germany; Puretex® surface). Three patients with each one implant with peri-implantitis were lost to follow-up (2 patients from the control group, 1 from the test group).

**Table 1:** Characteristics of included patients/implants

Characteristics	Control	Test
Number of patients	14	14
Age (years; mean [SD])	57.0 (13.7)	60.9 (7.2)
Gender; M (male), F (female)	M5, F9	M7, F7
Smoking; *n* subjects (%)	1 (7%)	3 (21%)
History of periodontitis; *n* subjects (%)	4 (29%)	5 (36%)
Dental status; *n* subjects (%)		
- Partially edentulous	13 (93%)	12 (86%)
- Fully edentulous	1 (7%)	2 (14%)
Total number of implants (range)	68 (1–9)	60 (1–10)
Number of implants with peri-implantitis (range)	22 (1–4)	31 (1–5)
Mean bone loss at baseline in mm (SD)	2.73 (1.49)	3.58 (1.57)

### Microbiological outcomes

^10^Log-transformed mean bacterial anaerobic counts of the culture-positive implants for the control and test group before and after debridement and decontamination of the implant surface during the surgical procedure are depicted in Table 2. In both groups, the debridement and decontamination procedure resulted in a significant immediate reduction in counts of anaerobic bacteria on the implant surface. Although the reduction in total anaerobic load was greater in the test group, the difference did not reach the level of statistical significance (*p* = 0.108). However, in the test group, the total anaerobic load was significantly more often reduced below detection level than in the control group (20 out of 23 in the test group, 10 out of 17 in the control group, *p* = 0.042). No significant differences were observed in the ^10^Log-transformed mean bacterial anaerobic counts of the peri-implant sulcus, neither between control and test group nor between baseline and 3 months after surgery (Table 3).

**Table 2:** Log-transformed mean bacterial anaerobic counts (SD) of culture-positive implants for the control and test group before (T_pre_) and after (T_post_) debridement and decontamination of the implant surface (intra-operative microbrush samples)

*N* = 40^a^	Total anaerobic bacterial load Log-transformed mean (SD)				
	T_pre_	T_post_	Difference	β (95% CI)^b^	*p* value
Control	5.57 (0.93) [17]	2.25 (2.98)^c^ [7]^d^	2.68 (3.25)	−1.39 (−3.09–0.32)	0.108
Test	5.35 (0.98) [23]	0.81 (2.25)^c^ [3]^d^	4.19 (3.31)		

*SD* standard deviation, *[n]* number of culture-positive implants

^a^Implants with baseline values of 0 excluded from analysis

^b^Linear regression analysis, adjusted for baseline values

^c^Significant difference from baseline

^d^Significant difference in number of culture-positive implants after decontamination between test and control group (*p* = 0.042)

**Table 3:** Log-transformed mean bacterial anaerobic counts (SD) for the control and test group before (T_0_) and 3 months after (T_3_) the surgical treatment (paperpoint samples)

*N* = 47^a^	Total anaerobic bacterial load Log-transformed mean (SD)				
	T_0_	T_3_	Difference	*β* (95% CI)^b^	*p* value
Control	6.69 (1.32)	6.31 (1.30)	0.38 (1.36)	−0.26 (−0.84–0.33)	0.377
Test	6.53 (1.06)	5.98 (0.94)	0.55 (0.99)		

*SD* standard deviation

^a^Three samples without bacterial growth and three samples without follow-up excluded from analysis

^b^Linear regression analysis, adjusted for baseline values

### Clinical outcomes

Descriptive statistics of the clinical outcomes at baseline and follow-up are depicted in Table 4. At 3-month follow-up, 75% of the implants (66.7% of the patients) in the control group and 63.3% of the implants (53.8% of the patients) in the test group showed no clinical signs of inflammation (PPD ≤4 mm without bleeding and/or suppuration on probing) (Table 4). The results from the multilevel analyses regarding the effects of the intervention on BoP, SoP, and PPD are shown in Table 5. No significant differences in BoP, SoP, and mean PPD were detected between control and test group at 3 months after surgery, neither in the “crude” nor in the “adjusted” analysis.

**Table 4:** Descriptive statistics of clinical parameters

		Control		Test	
		T_0_ (*n* = 22)	T_3_ (*n* = 20)	T_0_ (*n* = 31)	T_3_ (*n* = 30)
Plaque	% of sites (SD) % of implants (n)	4.5 (12.5) 13.6 (3)	10.0 (18.8) 25.0 (5)	4.0 (9.3) 16.1 (5)	2.5 (7.6) 9.7 (3)
BoP	% of sites (SD) % of implants (n)	86.4 (18.5) 100 (22)	28.8 (35.6) 50 (10)	66.1 (29.3) 96.8 (30)	39.2 (31.3) 76.7 (23)
SoP	% of sites (SD) % of implants (n)	22.7 (24.3) 54.5 (12)	5.0 (15.4) 10.0 (2)	30.7 (20.1) 80.6 (25)	8.3 (20.1) 20.0 (6)
Mean PPD	Mean (SD)	5.3 (1.1)	3.5 (1.5)	5.2 (1.1)	4.1 (1.6)
PPD ≥5 mm	% of sites (SD) % of implants (n)	67.1 (26.0) 100 (22)	18.8 (30.2) 35.0 (7)	61.3 (22.2) 100 (31)	28.3 (33.9) 46.7 (14)
PPD ≥6 mm	% of sites (SD) % of implants (n)	50.0 (27.8) 100 (22)	12.5 (26.3) 25.0 (5)	46.8 (26.4) 90.3 (28)	24.2 (33.1) 40.0 (12)
PPD ≥5 mm + BoP/SoP (same site)	% of sites (SD) % of implants (n) % of patients (n)	65.9 (26.2) 100 (22) 100 (14/14)	12.5 (25.0) 25.0 (5) 33.3 (4/12)	54.8 (22.7) 100 (31) 100 (14/14)	20.0 (29.7) 36.7 (11) 46.2 (6/13)
PPD ≥6 mm + BoP/SoP (same site)	% of sites (SD) % of implants (n) % of patients (n)	50.0 (27.8) 100 (22) 100 (14/14)	8.8 (20.3) 20.0 (4) 33.3 (4/12)	41.1 (24.6) 90.3 (28) 100 (14/14)	17.5 (28.7) 33.3 (10) 46.2 (6/13)

**Table 5:** Average differences in BoP, SoP, and PPD between the control and test group at 3-month follow-up

Outcome variable	Crude model^a^ β (95% CI)	*p* value	Adjusted model^b^ *β* (95% CI)	*p*-value
% Sites BoP % Sites SoP Mean PPD	16.2 (−7.9 to 40.3) 0.0 (−10.9 to 10.9) 0.6 (−0.6 to 1.8)	0.743 1.000 0.205	7.9 (−16.4 to 32.3) 0.7 (−10.1 to 11.4) 0.2 (−1.0 to 1.3)	0.821 0.882 0.470

The reference category for intervention effect is the control group. The regression coefficients (*β*) indicate the average differences in clinical outcomes between the control and test group at 3-month follow-up

*BoP* bleeding on probing, *SoP* suppuration on probing, *PPD* probing pocket depth, *95% CI* 95% confidence interval

^a^Adjusted for baseline values

^b^Adjusted for baseline values, smoking, and mean bone loss at baseline

## Discussion

This randomized controlled trial aimed to determine the effect of 35% phosphoric etching gel on decontamination of the implant surface during resective surgical treatment of peri-implantitis. Both decontamination procedures (mechanical debridement with curettes and gauzes combined with phosphoric acid 35% and mechanical debridement combined with sterile saline) resulted in a significant immediate reduction in counts of anaerobic bacteria on the implant surface. This immediate reduction was greater when phosphoric acid was used. Although the difference in log-transformed mean anaerobic counts between both decontaminating procedures did not reach the level of statistical significance (*p* = 0.108), there were significantly less culture-positive implants after the decontamination procedure in the phosphoric acid group (*p* = 0.042). As our study focused on the decontaminating effect of phosphoric acid on implant surfaces, we used the microbiological parameter as primary outcome variable. To evaluate the effect of the intervention on this microbiological parameter, an in vivo situation was chosen to benefit the influence of a clinical situation. In addition, we evaluated secondary outcome parameters indicating the clinical effect of the treatment procedure, i.e., disease resolution 3 months after active treatment.

At 3 months post-surgery, disease resolution was more frequently observed in the control group (75% of implants) than in the test group (63.3% of implants). However, no significant differences in clinical and microbiological outcomes between control and test group were found. Although the study was “a priori” not powered to detect clinical differences, no trend was observed for superior results of one decontamination procedure over the other.

To our knowledge, this is the first randomized controlled clinical trial reporting on the effect of phosphoric acid in relation to peri-implantitis treatment. The reason for choosing phosphoric acid as decontaminating agent was that acids with low pH exert a strong bactericidal effect [22, 36], and phosphoric acid does not seem to chemically damage titanium implant surface [37]. A gel as application mode has the great advantage of being precisely applicable with minimal touching of the surrounding bone or connective tissue. A disadvantage of a gel might be the limited flow in deeper areas of the rough implant surface. To overcome this problem, it was decided to continuously rub the etching gel onto the implant surface with a small brush during the decontamination period.

Phosphoric acid gel as agent for implant surface decontamination has only been investigated in two other clinical studies [26, 27]. Strooker et al. [26] used phosphoric acid 35% for peri-implant supportive therapy and found greater reductions in bacterial load, but no significant clinical differences compared to conventional mechanical supportive therapy. They concluded that local application of 35% phosphoric acid gel can be as effective as conventional mechanical therapy in the professional supportive care of oral implants. In the study of Wiltfang et al. [27], 20% etching gel was used for implant surface decontamination in a combined surgical protocol for treatment of peri-implantitis. Thirty-six implants with peri-implantitis in 22 patients were followed for 1 year. The implants were decontaminated with etching gel, and the defects were filled with autologous bone mixed with an osteoinductive material for regenerative treatment of bone defects. In their study, previous microbiological tests (not published) of implants in situ had revealed complete elimination of the bacterial microflora after decontamination with etching gel, which is close to our results of “complete” elimination (reduction below detection level) in 20 out of 23 implants. They concluded that their surgical protocol in combination with phosphoric etching gel provides a reliable method to treat peri-implant bone defects.

Phosphoric acid used in an in vitro setting has only been described in a study by Tastepe et al. [37]. The use of an air abrasive device with four different powders was compared to phosphoric acid. In contrast to our study and the previous described clinical studies, the use of phosphoric acid was not efficient in removing biofilm. The residual biofilm area was significantly greater after treatment with phosphoric acid compared to air abrasive treatment with powder or even control treatment without powder. Apparently, only water and air might be effective in reducing the biofilm. Nonetheless, when the titanium surface was viewed under a scanning electron microscopy (SEM), no visible titanium surface change was seen after phosphoric acid application while some minor changes (dependent on the character and size of the particles) were observed after air powder abrasive treatment.

Recent studies that zoom in on titanium surface physico-chemistry reveal interesting results [38, 39]. Kotsakis et al. [38] hypothesized that chemical residues alter the titanium surface physicochemistry and subsequently compromise cellular response to these decontaminated surfaces. However, they report on effective restoring of biocompatibility when sterile saline, citric acid, and EDTA/sodium hypochlorite (NaOCl-EDTA) were used, in contrast to chlorhexidine. Therefore, they propose the use of sterile saline, citric acid, and NaOCl-EDTA in the treatment of peri-implantitis not only for their antimicrobial properties but also for the preservation of the titanium material properties. In contrast, a study by [39] found noticeable morphological changes and corrosion on the titanium surface when the synergistic effect of acidic environments (i.e., citric acid, 15% hydrogen peroxide, tetracycline, peroxyacetic acid) and mechanical forces (rubbing with cotton swabs) was investigated. Dissolution of the oxide layer (which can result in corrosion) was observed when using peroxyacetic and citric acid. It is therefore hypothesized that surface damage of dental alloys may potentially be induced after detoxification and maintenance treatments with acidic solutions and subsequently might hinder re-osseointegration. No visibly evident damage of the surfaces was shown by [39] when neutral or basic treatments such as sodium fluoride 0.12, 0.20, and 1.10% were used, which might be explained by the neutral electrochemical environment [40].

Interpreting the results of these in vitro studies has to be done cautiously since the results among the studies are not homogenous and the effects of the chemical environment coupled with mechanical force in the oral environment has to be further evaluated. In our study, however, phosphoric acid neither seemed to have a positive nor a negative effect on clinical outcomes.

The current study is based on a follow-up time of 3 months and therefore the long-term results on the use of phosphoric acid remain unclear.

## Conclusions

Implant surface decontamination is considered a highly susceptible step in the treatment of peri-implantitis. The application of 35% phosphoric acid after mechanical debridement is superior to mechanical debridement combined with sterile saline rinsing for decontamination of the implant surface during surgical peri-implantitis treatment. However, phosphoric acid as implant surface decontaminant does not seem to enhance clinical outcomes on a 3-month follow-up. Larger studies with a longer follow-up period are needed to validate these findings.

## Abbreviations

GR: Gerry Raghoebar
DH: Diederik Hentenaar
YDW: Yvonne de Waal

## References

[1] Lang NP, Berglundh T, Working Group 4 of Seventh European Workshop on Periodontology (2011). Periimplant diseases: where are we now?—Consensus of the Seventh European Workshop on Periodontology. J Clin Periodontol.

[2] Derks J, Tomasi C (2015). Peri-implant health and disease. A systematic review of current epidemiology. J Clin Periodontol.

[3] Derks J, Schaller D, Håkansson J, Wennström JL, Tomasi C, Berglundh T (2016). Peri-implantitis—onset and pattern of progression. J Clin Periodontol.

[4] Lindhe J, Meyle J, Group D of European Workshop on Periodontology (2008). Peri-implant diseases: Consensus Report of the Sixth European Workshop on Periodontology. J Clin Periodontol.

[5] Leonhardt A, Dahlén G, Renvert S (2003). Five-year clinical, microbiological, and radiological outcome following treatment of peri-implantitis in man. J Periodontol.

[6] Máximo MB, de Mendonça AC, Renata Santos V, Figueiredo LC, Feres M, Duarte PM (2009). Short-term clinical and microbiological evaluations of peri-implant diseases before and after mechanical anti-infective therapies. Clin Oral Implants Res.

[7] Serino G, Turri A (2011). Outcome of surgical treatment of peri-implantitis: results from a 2-year prospective clinical study in humans. Clin Oral Implants Res.

[8] Bassetti M, Schär D, Wicki B, Eick S, Ramseier SA, Arweiler NB, Sculean A, Salvi GE (2014). Anti-infective therapy of peri-implantitis with adjunctive local drug delivery or photodynamic therapy: 12-month outcomes of a randomized controlled clinical trial. Clin Oral Implants Res.

[9] De Waal YC, Raghoebar GM, Meijer HJ, Winkel EG, van Winkelhoff AJ (2016). Prognostic indicators for surgical peri-implantitis treatment. Clin Oral Implants Res.

[10] De Waal YC, Raghoebar GM, Huddleston Slater JJ, Meijer HJ, Winkel EG, van Winkelhoff AJ (2013). Implant decontamination during surgical peri-implantitis treatment: a randomized, double-blind, placebo-controlled trial. J Clin Periodontol.

[11] Riben-Grundstrom C, Norderyd O, André U, Renvert S (2015). Treatment of peri-implant mucositis using a glycine powder air-polishing or ultrasonic device: a randomized clinical trial. J Clin Periodontol.

[12] Heitz-Mayfield LJA, Salvi GE, Mombelli A, Faddy M, Lang NP (2012). Anti-infective surgical therapy of peri-implantitis. A 12-month prospective clinical study. Clin Oral Implants Res.

[13] Esposito M, Grusovin MG, Worthington HV (2012). Treatment of peri-implantitis: what interventions are effective? A Cochrane systematic review. Eur J Oral Implantol.

[14] Louropoulou A, Slot DE, Van der Weijden F (2014). The effects of mechanical instruments on contaminated titanium dental implant surfaces: a systematic review. Clin Oral Implants Res.

[15] Ramanauskaite A, Daugela P, Faria de Almeida R, Saulacic N (2016). Surgical non-regenerative treatments for peri-implantitis: a systematic review. J Oral Maxillofacial Res.

[16] Schwarz F, Becker K, Bastendorf KD, Cardaropoli D, Chatfield D, Dunn I, Fletcher P, Einwag J, Louropoulou A, Mombelli A, Ower P, Pavlovic P, Sahrmann P, Salvi GE, Schmage P, Takeuchi Y, Van Der Weijden F, Renvert S (2015). Recommendations on the clinical application of air polishing for the management of peri-implant mucositis and peri-implantitis. Quintessence Int J Pract Dent.

[17] Subramani KK (2012). Decontamination of titanium implant surface and re-osseointegration to treat peri-implantitis: a literature review. Int. J. Oral Maxillofac. Implants.

[18] Gosau M, Hahnel S, Schwarz F, Gerlach T, Reichert TE, Bürgers R (2010). Effect of six different peri-implantitis disinfection methods on in vivo human oral biofilm. Clin Oral Implants Res.

[19] Heitz-Mayfield LJ, Salvi GE, Mombelli A, Faddy M, Lang NP (2012). Implant Complication Research Group. Anti-infective surgical therapy of peri-implantitis. A 12-month prospective clinical study. Clin Oral Implants Res.

[20] Meyle J (2012). Mechanical, chemical and laser treatments of the implant surface in the presence of marginal bone loss around implants. Eur J Oral Implantol.

[21] Ntrouka VI, Slot DE, Louropoulou A, Van der Weijden F (2011). The effect of chemotherapeutic agents on contaminated titanium surfaces: a systematic review. Clin Oral Implants Res.

[22] Chen CJ, Chen CC, Ding SJ (2016). Effectiveness of hypochlorous acid to reduce the biofilms on titanium alloy surfaces in vitro. Int J Mol Sci.

[23] Dennison DK, Huerzeler MB, Quinones C, Caffesse RG (1994). Contaminated implant surfaces: an in vitro comparison of implant surface coating and treatment modalities for decontamination. J Periodontol.

[24] Htet M, Madi M, Zakaria O, Miyahara T, Xin W, Lin Z, Aoki K, Kasugai S (2016). Decontamination of anodized implant surface with different modalities for peri-implantitis treatment: lasers and mechanical debridement with citric acid. J Periodontol.

[25] Mouhyi J, Sennerby L, Van Reck J (2000). The soft tissue response to contaminated and cleaned titanium surfaces using CO2 laser, citric acid and hydrogen peroxide. An experimental study in the rat abdominal wall. Clin Oral Implants Res.

[26] Strooker H, Rohn S, Van Winkelhoff AJ (1998). Clinical and microbiologic effects of chemical versus mechanical cleansing in professional supportive implant therapy. Int. J. Oral Maxillofac. Implants.

[27] Wiltfang J, Zernial O, Behrens E, Schlegel A, Wamke PH, Becker ST (2012). Regenerative treatment of peri-implantitis bone defects with a combination of autologous bone and a demineralized xenogenic bone graft: a series of 36 defects. Clin Implant Dent Relat Res.

[28] Wohlfahrt JC, Lyngstadaas SP, Rønold HJ, Saxegaard E, Ellingsen JE, Karlsson S, Aass AM (2012). Porous titanium granules in the surgical treatment of peri-implant osseous defects: a randomized clinical trial. Int. J. Oral Maxillofac. Implants.

[29] Zablotsky MH, Diedrich DL, Meffert RM (1992). Detoxification of endotoxin-contaminated titanium and hydroxyapatite-coated surfaces utilizing various chemotherapeutic and mechanical modalities. Implant Dent.

[30] Alhag M, Renvert S, Polyzois I, Claffey N (2008). Re-osseointegration on rough implant surfaces previously coated with bacterial biofilm: an experimental study in the dog. Clin Oral Implants Res.

[31] Kolonidis SG, Renvert S, Hämmerle CH, Lang NP, Harris D, Claffey N (2003). Osseointegration on implant surfaces previously contaminated with plaque. An experimental study in the dog. Clin Oral Implants Res.

[32] De Waal YC, Raghoebar GM, Meijer HJ, Winkel EG, van Winkelhoff AJ (2015). Implant decontamination with 2% chlorhexidine during surgical peri-implantitis treatment: a randomized, double-blind, controlled trial. Clin Oral Implants Res.

[33] Syed SA, Loesche WJ (1972). Survival of human dental plaque flora in various transport media. Appl Microbiol.

[34] Van Winkelhoff AJ, van Steenbergen TJ, Kippuw N, De Graaff J (1985). Further characterization of Bacteroides endodontalis, an asaccharolytic black-pigmented Bacteroides species from the oral cavity. J Clin Microbiol.

[35] Zambon JJ (1996). Periodontal diseases: microbial factors. Ann Periodontol.

[36] Héritier M (1984). Effects of phosphoric acid on root dentin surface. A scanning and transmission electron microscopic study. J Periodontal Res.

[37] Tastepe CS, Lui Y, Visscher CM, Wismeijer D (2013). Cleaning and modification of intraorally contaminated titanium discs with calcium phosphate powder abrasive treatment. Clin Oral Implants Res.

[38] Kotsakis GA, Lan C, Barbosa J, Lill K, Chen R, Rudney J, Aparicio C (2016). Antimicrobial agents used in the treatment of peri-implantitis alter the physicochemistry and cytocompatibility of titanium surfaces. J Periodontol.

[39] Wheelis SE, Gindri IM, Valderrama P, Wilson TG, Huang J, Rodrigues DC (2016). Effects of decontamination solutions on the surface of titanium: investigation of surface morphology, composition, and roughness. Clin Oral Implants Res.

[40] Suito H, Iwawaki Y, Goto T, Tomotake Y, Ichikawa T (2013). Oral factors affecting titanium elution and corrosion: an in vitro study using simulated body fluid. PLoS One.

